# Using a Quality-Driven Approach to Maintain an N-95 Respirator Supply During a Pandemic-Driven Global Shortage

**DOI:** 10.1017/ash.2021.92

**Published:** 2021-07-29

**Authors:** Amy Selimos, Mark Buchanan, Lauren DiBiase, Stephen Dean, Pat Boone, Nicholas Shaheen, Emily Sickbert-Bennett Vavalle, Beth Willis

## Abstract

**Background:** Reports of hospitals overwhelmed by COVID-19 patients created severe shortages of personal protective equipment (PPE). In this large academic medical system, we used a systematic team approach to proactively maintain an adequate PPE supply. The team consisted of staff from multiple departments including infection prevention, environmental health and safety, operational efficiency, and supply chain. The healthcare system solicited donations of PPE, and our team was tasked with developing a sustainable method to provide healthcare workers with safe and effective N-95 respirators. Respirators are normally fitted to our 6,000+ healthcare workers through a fit-testing process using 4 models of N-95s. We received >60 models, many in small quantities, posing a new level of complexity that prevented use of our typical fit-testing method. **Methods:** Donated respirators were manually verified on the CDC/NIOSH website to validate approval or approved alternative. A categorization system was developed, and respirators were sorted based on quality, style, and condition. User seal checks replaced qualitative fit testing due to the uncertain and quickly changing respirator supply. Staff were educated about the importance of performing a seal check to evaluate respirator fit and were provided instructions for what to do if they failed a seal check. We performed limited quantitative fit testing on a small group previously fit tested to 1 of the 4 models of N-95s normally stocked to identify the most effective alternative respirators to serve as substitute N-95s. **Results:** We were able to provide staff with new N-95s and delay the release of reprocessed N-95s. Overall, 18 models of respirators were tested on staff for filtration effectiveness and fit. We deemed 61% masks to be of last resort, and these were not released. We determined that 39% were acceptable as an alternative for at least 1 of our usual respirator models. However, only 3 models (17%) available in small quantities fit wearers whose size was in shortest supply. This scarcity led to the evaluation and purchase of a new respirator prototype for small N-95 wearers, which was an important success of our team’s work and for staff safety. **Conclusions:** Collaboration between teams from a variety of backgrounds, using both qualitative and quantitative data, resulted in a sustainable method for receiving, sorting, and evaluating donated N-95 respirators, ensuring the delivery of a steady supply of effective N-95 respirators to our staff. This quality-driven approach was an efficient and effective strategy to maintain our N-95 respirator supply during a pandemic driven global shortage.

**Funding:** No

**Disclosures:** None

